# Burden of Neurological Diseases in the Philippines as Revealed by Web Searches: An Infodemiological Study

**DOI:** 10.3390/ijerph192416736

**Published:** 2022-12-13

**Authors:** Anisah Hayaminnah D. Alonto, Almira Doreen Abigail O. Apor, Roland Dominic G. Jamora

**Affiliations:** Department of Neurosciences, College of Medicine and Philippine General Hospital, University of the Philippines Manila, Manila 1000, Philippines

**Keywords:** neurological diseases, Philippines, infodemiology, web searches, bid values

## Abstract

Due to the real-time acquisition of big data from the Internet, analysis of Google queries is now recognized as a valuable tool to explore and predict human behavior and interests. It was suggested that online data can be correlated with actual health data. Although the data are not structured nor systematic, the huge data from search engines can easily identify trends concerning diseases and other health concepts from a population perspective. Moreover, Internet data with the use of web search advertising nowadays may not only reveal the interest of the general population but also of the healthcare industry as reflected by the bid prices in search terms for medications. We aimed to compare the interests of the general population using monthly search volumes from Google and the healthcare industry using bid prices in web searches. Data used in this study were obtained from the Google Ads Application Programming Interface (API). This study evaluated the population’s interest in neurological disorders by using search volumes related to neurology, either disease diagnosis or medications. Bid values generated in API were used as a proxy for the interests of the healthcare industry. Spearman’s rank-order correlation was performed between search volumes and bid prices to determine significance. Among the neurologic diseases listed, the most searched were attention deficit hyperactivity disorder, migraine, and Alzheimer’s disease. The most commonly searched drugs were oral antihypertensives (amlodipine, losartan, carvedilol), lipid-lowering agents (atorvastatin, simvastatin, rosuvastatin), and antiplatelets (acetylsalicylic acid, clopidogrel). The other most searched drugs were analgesics such as acetaminophen, tramadol, diclofenac, and morphine. The correlational analysis did not reveal a statistically significant correlation between search volume and bid price for both neurologic diseases and medications. Web searches may reflect the interest of the general population and the healthcare industry. However, there was disagreement in the search interests of the general population and the scientific community, including the pharmaceutical industry. Further studies are necessary in order to align these interests for the common benefit of all stakeholders.

## 1. Introduction

Neurological disorders are the foremost cause of disability and the second leading cause of death globally [1]. The increasing burden of deaths and disabilities caused by neurological disorders is now recognized as a global public health challenge, with the greatest burden in low-income and middle-income countries [1]. In 2019, the disability-adjusted life-years (DALY) of neurological disorders were 64.6 million in South-East Asia and 85.0 million in western Pacific regions, with the highest DALYs observed for stroke and Alzheimer’s disease (AD) and other types of dementia [2]. In the Philippines, however, there is no available data on the prevalence of neurological diseases in the country.

With the increasing use of the Internet for health-related purposes [3,4,5] and the real-time acquisition of fast and big data, analysis of Google queries is now recognized as a valuable tool to explore and predict human behavior and interests, hence thought to be useful in health information [6]. It was suggested that online data can be correlated with actual health data [6]. With the use of the Internet and infodemiological studies, the distribution and tracking of health information in a population can now be measured, possibly leading to reliable and meaningful indicators to track health information supply and demand [7]. Although the data are not structured nor systematic, the huge data from search engines can easily identify frequencies and trends in diseases and other health concepts from a population perspective [8,9,10,11,12,13,14]. Patients with neurological diseases are some of the most active on the Internet and many of them utilize digital healthcare [15], likely due to the disability incurred from their neurological condition, which limits their mobility. Moreover, Internet data with the use of web search advertising nowadays may not only reveal the interest of the general population but also the interests of the healthcare industry as reflected by the bid prices in search terms for medications.

In this study, we aimed to analyze the searches related to neurological diseases and nervous system drugs in the Philippines in order to infer the interests of the general population and the healthcare industry from Google search volumes and bid prices.

## 2. Materials and Methods

### 2.1. Web Searches

We analyzed the monthly searches from Google, the most commonly used search engine in the country [16]. All data in this study were obtained from Google Ads Application Programming Interface (API). The aggregate data from July 2021 to June 2022, the longest time duration allowed by the API, were obtained. Due to the lack of formal Filipino translation of neurological diseases [17] and more frequent use of English terms in the Philippine hospital setting, only searches carried out in English were analyzed.

### 2.2. Databases

This study used the list of diseases from the National Institute of Neurological Disorders and Stroke, which provided a list of 448 neurological diseases. Common disease abbreviations were added to the list to enrich the search. In addition, this study listed the drugs used for neurological disorders from the 2019 Philippine National Formulary, including generic and brand names, leading to a list of 463 drugs. Search volumes of brand-name drugs were aggregated with their generic drug counterpart.

### 2.3. Analysis

This study evaluated the population’s interest in neurological disorders by using search volumes related to neurology, either disease diagnosis or medications. Frequency or search volume of a query can be a good proxy for the interest of the general population, specifically Internet users. Similarly, real-time bids for web queries by advertisers may evaluate the interests of the healthcare industry. In search advertising, an auction is performed for each query, where for each click from an Internet user the advertiser pays for what the next advertiser is willing to pay [15]. Therefore, the bid values generated in API are a good proxy for the interests of the healthcare industry (advertiser). For this study, the standard advertising API of Google which is restricted to the Philippine market was used to evaluate the interests of the healthcare industry in the country. Spearman’s rank-order correlation was performed to determine association between the average monthly search volume and average bid. Statistical significance was set at *p* < 0.05.

## 3. Results

### 3.1. Frequency of Neurological Diseases in Web Searches

**Figure 1:**
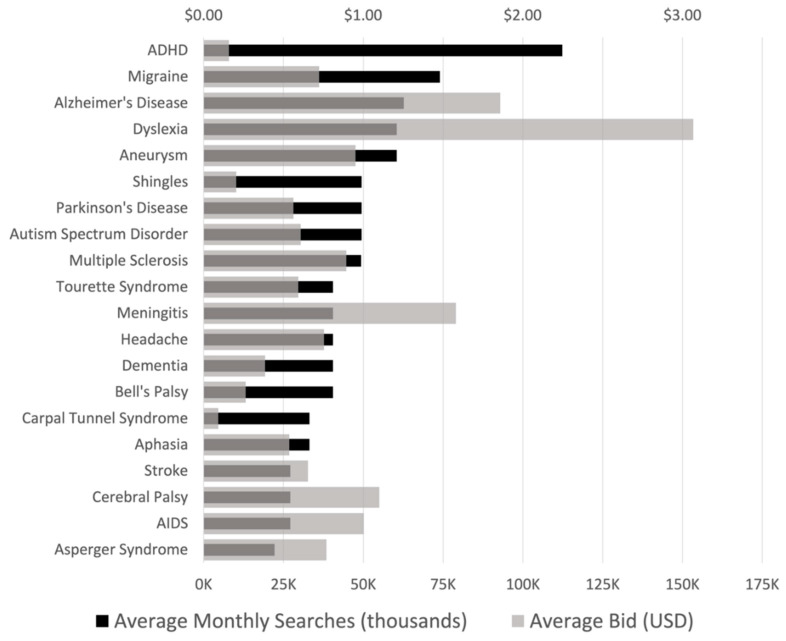
Average monthly search volumes and bid prices of top 20 neurological diseases in the Philippines. (USD 1.00 = PHP 55.87 as of July 2022).

The interest of Internet users in neurological diseases was evaluated using the search volume of terms related to neurological diseases (full name or common abbreviations) in Google. Among the neurologic diseases listed, the most searched were attention deficit hyperactivity disorder (ADHD), migraine, and AD (Figure 1).

In the 2019 global burden of neurological disorders, ADHD was not included in the most prevalent neurologic disorders. However, in this study, ADHD was noted to be the most searched among neurological diseases. Tourette syndrome, which is also not part of the common neurologic disorders worldwide, was also seen to be one with a high search volume.

Although belonging to the top 20 most searched diseases, stroke, the most prevalent neurological disease [1], surprisingly did not have the highest search volume. Migraine, AD, Parkinson’s disease (PD), multiple sclerosis (MS), and meningitis were noted to be both seen with high search volume and among the most prevalent neurological diseases in the 2019 global burden of neurological diseases [1].

The average bids were also analyzed for the neurologic diseases, which showed the highest bid for dyslexia, AD, and meningitis (Figure 1).

### 3.2. Frequency of Central Nervous System (CNS) Drugs Searches

**Figure 2:**
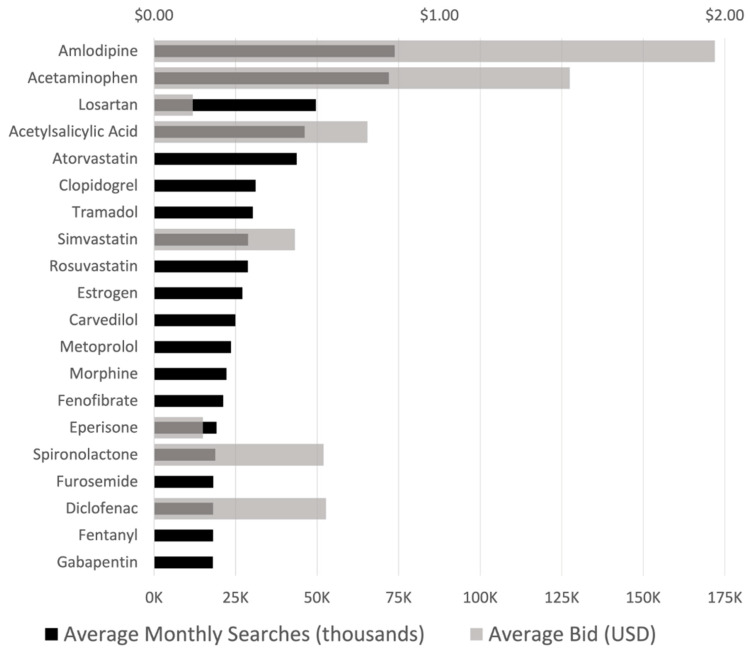
Average monthly search volumes and bid prices of top 20 drugs used for neurological diseases in the Philippines. (USD 1.00 = PHP 55.87 as of July 2022).

The most commonly searched drugs were oral antihypertensives (amlodipine, losartan, carvedilol), lipid-lowering agents (atorvastatin, simvastatin, rosuvastatin), and antiplatelets (acetylsalicylic acid, clopidogrel) (Figure 2). The other most searched drugs were analgesics such as acetaminophen, tramadol, diclofenac, and morphine.

Average bids were seen to be high for medications that are usually maintained for stroke patients such as amlodipine, acetylsalicylic acid, and simvastatin. Among the analgesics, the drugs with the highest bids were over-the-counter medications such as acetaminophen and diclofenac.

Although the search volumes of migraine, AD, PD, and MS were noted to be high, medications for these diseases were not the most searched drugs nor found to have the most bids.

### 3.3. Analysis Based on Web Searches

**Figure 3:**
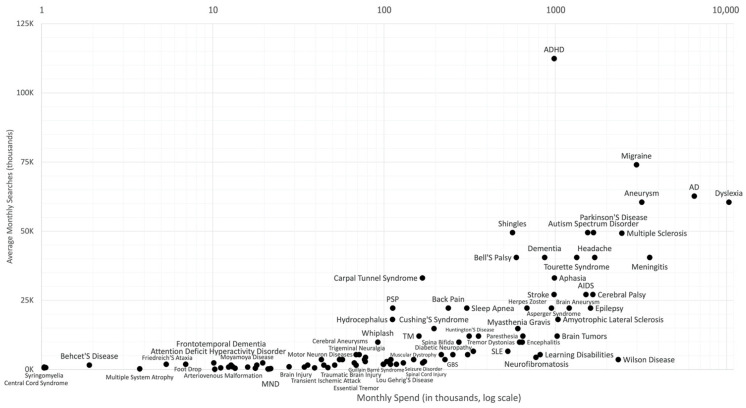
Relationship of query volume and advertising expenditure (ad bid) for neurological diseases searches. *Y*-axis shows average monthly searches and the suggested ad bid in the *X*-axis (in log scale, USD). In each graph, queries that are at the top represent the Internet users’ interest while the queries that are on the right side represent the health industry’s interest. (USD 1.00 = PHP 55.87 as of July 2022).

The relationship between query volume and query bid (value per click) was analyzed. Migraine, brain aneurysm, and dyslexia were ranked high in both dimensions (Figure 3).

**Figure 4:**
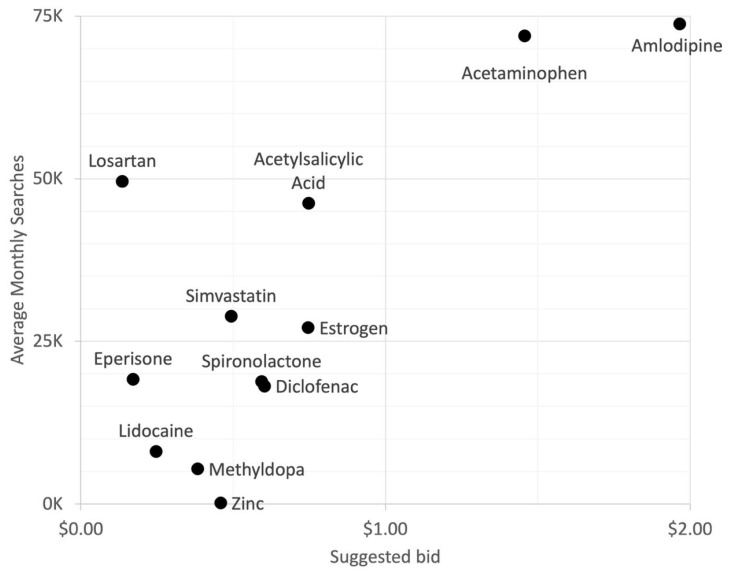
Relationship of query volume and advertising expenditure (ad bid) for central nervous system drugs searches. *Y*-axis shows average monthly searches and the suggested ad bid in the *X*-axis (in USD). In each graph, queries that are at the top represent the Internet users’ interest while the queries that are on the right side represent the health industry’s interest. (USD 1.00 = PHP 55.87 as of July 2022).

“AD”, which is an abbreviation for Alzheimer’s disease, was also noted to have ranked high. However, “AD” may also refer to “advertisement”, hence this could be falsely high. Among those with high search volumes were diseases that are commonly diagnosed in the younger population such as dyslexia, ADHD, and migraine. This was likely due to Internet use being more prevalent among the younger demographic [18]. For CNS drugs, acetaminophen and amlodipine ranked high in both dimensions (Figure 4).

Correlational analysis was also performed between the average monthly search volume and average bid for neurological diseases, showing a very weak correlation; however, this was not statistically significant (R = 0.14732, *p* = 0.14562). For neurological drugs, there was a moderate degree of correlation; however, this was also not statistically significant (R = 0.48951, *p* = 0.10625).

## 4. Discussion

This study evaluated the interest of Internet users in the Philippines on neurological diseases by analyzing the frequency of web searches on Google. Stroke, which is the most prevalent neurological disease [1], only had 27,100 average monthly searches. Meanwhile ADHD, the most searched neurological disease, had an average monthly search of 110,000. This was likely because ADHD is one of the most popular health-related hashtags in social media and awareness videos regarding it had been rampant on the Internet, where some users seek diagnosis after watching some videos about it on different platforms [19]. This can also be noted with Tourette Syndrome, which is not a common neurological disease but had an average monthly search of 40,500. Over the last few years, there has been a marked increase in tic-like behaviors after the consumption of videos on platforms such as TikTok or YouTube, that were mistakenly associated with Tourette Syndrome [20]. Therefore, findings in the average monthly searches of neurological diseases in this study were influenced not just by the prevalence of the diseases but also by social media trends, which raises awareness among the general population regarding these uncommon diseases. The other most commonly searched neurological diseases were also among the most prevalent diseases worldwide such as migraine, AD, aneurysm, PD, MS, and meningitis. Surprisingly, epilepsy, which is one of the more prevalent neurological diseases, was not part of the most searched neurological disease. This may reflect a lack of awareness among the general population [21].

It would also seem that there was disagreement in the search interest of laypersons and the scientific community. Among the neurologists in the country, the most commonly researched and published articles were about stroke and movement disorders [22]. There were significantly fewer published studies on headaches and neurodevelopmental and neuropsychiatric disorders [22], which were among the most searched neurological diseases on Google in this study. On the other hand, the interests of the pharmaceutical industry, as demonstrated by bid prices, may closely mirror that of the scientific community. Average bid prices were highest for medications used for secondary stroke prevention, which was reflective of stroke and cerebrovascular diseases being the most commonly researched disease in the Philippines [22]. This, however, may still be attributed to the general prevalence of lifestyle diseases and their underlying risk factors.

Among the medications, the most commonly searched were anti-hypertensives, analgesics, statins, and antithrombotics. Amlodipine (generic and brand names) had an average monthly search volume of 73,790. Other medications used for secondary stroke prevention were also among the most searched, such as losartan, acetylsalicylic acid, atorvastatin, and clopidogrel. These medications are available for free in local health units as part of the Philippine government’s campaign on treating non-communicable diseases such as stroke [23]. The high search volume for these drugs possibly reflects the vast number of people taking the said medications as secondary stroke prevention or even for other non-communicable diseases that is not necessarily neurologic. Analgesics, such as acetaminophen and tramadol, also had a high average monthly search volume.

Although neurological diseases such as AD, PD, and MS were seen to be one of the most searched, medications particular to these said diseases such as immunosuppressants, cholinesterase inhibitors, N-methyl-D-aspartate receptor antagonists, dopamine agonists, monoamine oxidase B and catechol-O-methyl transferase inhibitors were not found among the most searched drugs. This was likely because these are expensive drugs that are either not readily accessible or locally available [24,25], hence they are not very familiar to most Internet users and laypersons in general or are not being prescribed by their neurologists. This may also be reflective of the awareness of the general population regarding these diseases. In this regard, the lack of health education may also lead those with poor-health-seeking behavior to tend to self-diagnose instead [26] (e.g., all persons with cognitive decline are generalized as having AD), hence, the high search volume of some neurological diseases but not the medications particular to it. Measures for public health education may be implemented to correct prevailing public misconceptions.

In comparison to the study carried out in a developed country, the most commonly searched neurological diseases in the United States (US) were almost the same as with this study, but unique to the US list were shingles, sleep apnea, amyotrophic lateral sclerosis (ALS), and Bell’s palsy [15]. Sleep apnea and ALS are diseases that would require extensive workup, which is not very accessible to Filipino patients because of the out-of-pocket healthcare spending in the country [25]. The more commonly searched medications in the US were opioid analgesics (such as oxycodone, morphine, and hydrocodone) and antiseizure medications (such as gabapentin, clonazepam, and topiramate).

Several limitations should be considered in the interpretation of the data. First, these web searches may have only covered the Filipino population with access to the Internet, which is 70% of the population [18], and may not be considered representative of overall population interests. Nevertheless, to our knowledge, this was the first infodemiological study on the burden of neurological diseases carried out in a developing country. Although influenced by social media trends, this study may be a good proxy for the frequency of neurological diseases in the Philippines, for which the actual data are still unknown. Second, search strategies may differ between Internet users due to the use of abbreviations, synonyms, or colloquial expressions. However, in this study, only disease terms in English and their abbreviations were used for analysis due to the lack of Filipino translation of neurological diseases and the more frequent use of English terms in the Philippine hospital setting [17]. Lastly, this study only completed analysis limited to a single year, due to restrictions from the API. However, the analysis may be performed on a yearly basis in order to reveal trends in the frequency or interest in neurological diseases.

Epidemiological studies on neurologic diseases in the Philippines are still recommended to more accurately determine the prevalence of these diseases. This will better guide the scientific community and healthcare industry towards activities that will be beneficial to all stakeholders in the community—patients, providers, and the pharmaceutical industry.

## 5. Conclusions

This study assessed the interest of Internet users in the Philippines on neurological diseases. The most searched neurological diseases in the Philippines over the past year were ADHD, migraine, and AD. This may reflect the prevalence of these diseases in the country but this may also be highly influenced by social media trends on the Internet such as awareness campaigns and videos. The most commonly searched medications were anti-hypertensives, analgesics, statins, and antithrombotics, possibly reflecting the high number of people taking these drugs. Web searches may reflect the interest of Internet users and the healthcare industry. Nonetheless, our results showed disagreement in the search interests of Internet users and the scientific community, including the pharmaceutical industry. Further studies are necessary in order to align these interests for the common benefit of all stakeholders.

## Data Availability

The raw data supporting the conclusions of this article will be made available by the authors upon reasonable request.

## References

[1] Feigin V.L., Vos T., Nichols E., Owolabi M.O., Caroll W.M., Dichgans M., Deuschl G., Parmar P., Brainin M., Murray C. (2020). The global burden of neurological disorders: Translating evidence into policy. Lancet Neurol..

[2] Chang Y., Song T. (2022). The burden of neurological diseases in Asia: An analysis for the global burden of disease study 2019. Stroke.

[3] Bujnowska-Fedak M.M., Waligóra J., Mastalerz-Migas A. (2019). The internet as a source of health information and services. Adv. Exp. Med. Biol..

[4] Bilgin N., Kesgin M., Gücük S., Ak B. (2019). Assessment of internet usage for health-related information among clients utilizing primary health care services. Niger. J. Clin. Pract..

[5] Bujnowska-Fedak M.M. (2015). Trends in the use of the Internet for health purposes in Poland. BMC Public Health.

[6] Mavragani A., Ochoa G. (2019). Google trends in infodemiology and infoveillance: Methodology framework. JMIR Public Health Surveil..

[7] Eysenbach G. (2006). Infodemiology: Tracking flu-related searches on the web for syndromic surveillance. AMIA Annu. Symp. Proc..

[8] Alonto A.H.D., Jamora R.D.G., Leochico C.F.D., Espiritu A.I. (2022). Low online search interest in teleneurology before and during COVID-19 pandemic: An infodemiological study. Neurol. Sci..

[9] Moalong K.M.C., Jamora R.D.G., Roberto K.T., Espiritu A.I. (2021). Patterns of Google search behavior for epilepsy and seizures in the Philippines: An infodemiological study. Epilepsy Behav..

[10] Piamonte B.L.C., Anlacan V.M.M., Jamora R.D.G., Espiritu A.I. (2021). Googling Alzheimer disease: An infodemiological and ecological study. Dement. Geriatr. Cogn. Disord. Extra.

[11] Roberto K.T., Jamora R.D.G., Moalong K.M.C., Espiritu A.I. (2022). Infodemiology of autoimmune encephalitis, autoimmune seizures, and autoimmune epilepsy: An analysis of online search behavior using Google Trends. Epilepsy Behav..

[12] Layug E.J., Espiritu A.I., Calotes-Castillo L.V., Jamora R.D.G. (2021). The association of online search interest with polio cases and vaccine coverage: An infodemiological and ecological study. Eur. J. Pediatr..

[13] Perez J.A.L.S., Espiritu A.I., Jamora R.D.G. (2021). Google search behavior for meningitis and its vaccines: An infodemiological study. BMC Neurol..

[14] Mondia M.W.L., Espiritu A.I., Jamora R.D.G. (2022). Brain tumor infodemiology: Worldwide online health-seeking behavior using Google Trends and Wikipedia Pageviews. Front. Oncol..

[15] Baeza-Yates R., Sangal P.M., Villoslada P. (2017). Burden of neurological diseases in the US revealed by web searches. PLoS ONE.

[16] (2022). Digital Marketing for Asia. The Most Popular Search Engines across APAC. https://www.digitalmarketingforasia.com/the-most-popular-search-engines-across-apac/.

[17] Casanova-Gutierrez J. (2012). Epilepsy in Philippine language and dialects. Neurol. Asia.

[18] Schumacher S., Kent N. (2022). 8 Charts on Internet Use around the World as Countries Grapple with COVID-19. https://www.pewresearch.org/fact-tank/2020/04/02/8-charts-on-internet-use-around-the-world-as-countries-grapple-with-covid-19/.

[19] Yeung A., Ng E., Abi-Jaoude E. TikTok and attention-deficit/hyperactivity disorder: A cross-sectional study of social media content quality. Can. J. Psychiatry.

[20] Paulus T., Bäumer T., Verrel J., Weissbach A., Roessner V., Beste C., Münchau A. (2021). Pandemic tic-like behaviors following social media consumption. Mov. Disord..

[21] Moalong K.M.C., Espiritu A.I., Fernandez M.L.L., Jamora R.D.G. (2021). Treatment gaps and challenges in epilepsy care in the Philippines. Epilepsy Behav..

[22] Apor A.D.A.O., Jamora R.D.G. (2022). Research productivity among Filipino neurologists associated with socioeconomic, healthcare, and disease burden factors: A bibliometric analysis. Int. J. Environ. Res. Public Health.

[23] Department of Health (2022). Lifestyle-Related Diseases. https://doh.gov.ph/lifestyle-related-diseases..

[24] Ignacio K.H.D., Espiritu A.I., Jamora R.D.G. (2020). The current status and challenges in multiple sclerosis management in the Philippines. Mult. Scler. Relat. Disord..

[25] Jamora R.D.G., Miyasaki J.M. (2017). Treatment gaps in Parkinson’s disease care in the Philippines. Neurodegener. Dis. Manag..

[26] Aboueid S., Liu R.H., Desta B.N., Chaurasia A., Ebrahim S. (2019). The use of artificially intelligent self-diagnosing digital platforms by the general public: Scoping review. JMIR Med. Inform..

